# Epithelial-to-Mesenchymal Transition in the Pathogenesis and Therapy of Head and Neck Cancer

**DOI:** 10.3390/cancers9070076

**Published:** 2017-07-03

**Authors:** Julia Thierauf, Johannes Adrian Veit, Jochen Hess

**Affiliations:** 1 Section Experimental and Translational Head and Neck Oncology, Department of Otorhinolaryngology, Head and Neck Surgery, University Medical Center Heidelberg, Im Neuenheimer Feld 400, Heidelberg 69120, Germany; j.hess@dkfz-heidelberg.de; 2 Department of Otorhinolaryngology, Head and Neck Surgery, University Medical Center Ulm, Ulm 89075, Germany; johannes.veit@uniklinik-ulm.de; 3 Research Group Molecular Mechanisms of Head and Neck Tumors, German Cancer Research Center (DKFZ), Heidelberg 69120, Germany

**Keywords:** epithelial-to-mesenchymal transition, mesenchymal-to-epithelial transition, head and neck cancer, biomarkers

## Abstract

Head and neck cancer (HNC) is one of the most prevalent human malignancies worldwide, with a high morbidity and mortality. Implementation of interdisciplinary treatment modalities has improved the quality of life, but only minor changes in overall survival have been achieved over the past decades. Main causes for treatment failure are an aggressive and invasive tumor growth in combination with a high degree of intrinsic or acquired treatment resistance. A subset of tumor cells gain these properties during malignant progression by reactivating a complex program of epithelia-to-mesenchymal transition (EMT), which is integral in embryonic development, wound healing, and stem cell behavior. EMT is mediated by a core set of key transcription factors, which are under the control of a large range of developmental signals and extracellular cues. Unraveling molecular principles that drive EMT provides new concepts to better understand tumor cell plasticity and response to established as well as new treatment modalities, and has the potential to identify new drug targets for a more effective, less toxic, and individualized therapy of HNC patients. Here, we review the most recent findings on the clinical relevance of a mesenchymal-like phenotype for HNC patients, including more rare cases of mucosal melanoma and adenoid cystic carcinoma.

## 1. Head and Neck Cancer

Head and neck cancer (HNC) originates from the mucosal epithelia of the upper aero-digestive tract, and in the majority of cases is diagnosed as head and neck squamous cell carcinoma (HNSCC) of the oral cavity, the naso-, oro- or hypopharynx, the larynx, the paranasal sinuses, or nasal cavity [1]. Apart from tobacco and alcohol abuse, infection with high-risk types of human papilloma virus (HPV—in particular HPV16—has been established as an additional risk factor with a rising incidence in oropharyngeal SCC (OPSCC) [2]. HPV-related OPSCCs represent a distinct tumor entity with regard to cellular and molecular features and exhibit a favorable survival as compared to their HPV-negative counterparts [3].

Despite the implementation of interdisciplinary treatment modalities and improvements in early detection, surgical techniques, radiation therapy protocols, and chemotherapeutic regimes, the overall survival of advanced HNSCC has only marginally improved over the past decades and appropriate treatment remains a major challenge [4]. This is mainly due to the aggressive and invasive growth pattern as well as high resistance against available therapies, leading to loco-regional relapse and/or distant metastasis [5].

Mucosal melanoma (MM) and adenoid cystic carcinoma (ACC) of salivary glands belong to rare cases of head and neck cancer. MM originates from melanocytes of mucosal epithelia and makes up to 1% of all melanomas [6]. However, more than half of all cases can be found in the head and neck region (MMHN). There is compelling evidence highlighting molecular and clinical differences between cutaneous and mucosal melanoma in terms of tumor growth, metastasis, and pathogenesis [7]. MMHN is characterized by an infiltrative and local destructive growth pattern, and overall survival in these patients is more often limited by local recurrences rather than by distant metastases [8].

ACC originates from epithelial cells of the major or minor salivary glands of the head and neck, and is the second most common cancer of the salivary glands [9]. ACC is a neurotropic tumor with an infiltrative growth pattern preferentially along nerve fibers and a high tendency to local spread and early distant metastases, limiting therapeutic options with a curative intent of treatment [9].

## 2. Epithelial-to-Mesenchymal Transition in Cancer

**Figure 1:**
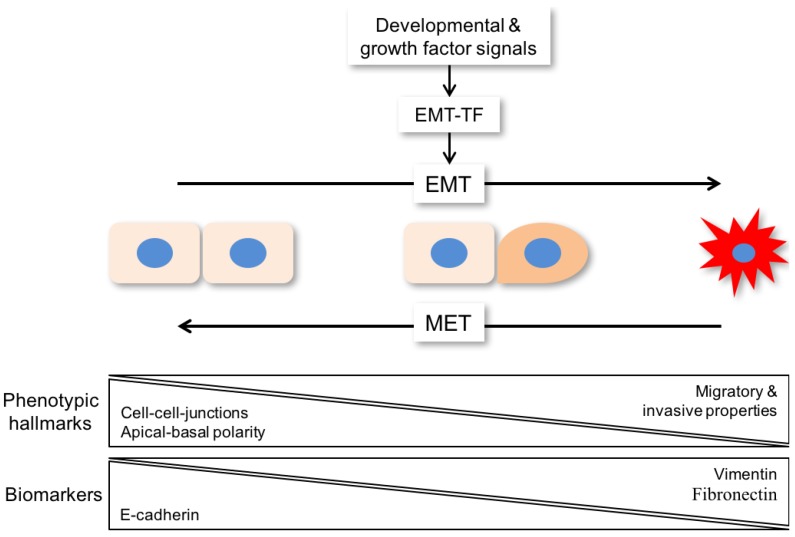
Scheme of EMT (epithelial-to-mesenchymal transition) as a program with dynamic transitional states, which are characterized by metastable intermediates. For metastatic colonization, a reversal of EMT known as mesenchymal-to-epithelial transition (MET) supports tumor cell expansion as an important prerequisite for metastatic growth. Phenotypic hallmarks of EMT are loss of cell-cell junctions, loss of apical-basal polarity, and acquisition of migratory and invasive properties. Those changes are induced by loss of E-cadherin and increased levels of biomarkers like vimentin and fibronectin. EMT-TF: EMT-transcription factor.

Epithelial-to-mesenchymal transition (EMT) is a complex process in which epithelial cells lose their characteristic features and acquire a mesenchymal-like phenotype [10]. Phenotypic hallmarks of EMT are the loss of cell-cell junctions, loss of apical-basal polarity, and acquisition of migratory and invasive properties (Figure 1). It resembles a fundamental process in embryonic development and during wound healing. In a pathophysiologic context, cancer cells are able to reactivate the EMT program to gain new properties such as accelerated motility and treatment resistance [11].

A large range of developmental and growth factor signals can drive EMT by triggering genetic and epigenetic programs, which are under the control of or regulate a core set of transcription factors (EMT-TF) belonging to different families, including SNAI1/2, TWIST1, and ZEB1/2, among others (Figure 1) [12]. In the past, most studies relied on morphological alterations complemented by the detection of epithelial (e.g., E-cadherin) and mesenchymal markers (e.g., vimentin, fibronectin, N-cadherin) to assess an epithelial or a mesenchymal state as a simple binary decision (Table 1). However, more recent evidence has advanced and broadened the definition of EMT as a program with dynamic transitional states characterized by metastable intermediates (Figure 1) [13]. This concept is in line with the high degree of tumor cell plasticity, which has been described for most tumor entities (including HNC), and might be the main driver of intrinsic or acquired resistance to established treatment regimens like platinum-based chemotherapy or radiation. 

Epithelial plasticity and mesenchymal conversion is often seen in cancer cells as they leave the primary tumor and disseminate to other parts of the body to colonize distant organs and form metastases, which is responsible for the vast majority of cancer-related deaths [13]. However, EMT-related invasion in combination with tumor cell dissemination is initiating, but is not sufficient for completion of the metastatic cascade [14]. For metastatic colonization, a reversal of EMT known as mesenchymal-to-epithelial transition (MET) supports tumor cell expansion as an important prerequisite for metastatic growth (Figure 1). In contrast to EMT, extracellular signals or cell intrinsic programs involved in the induction of MET have not been well characterized [13]. It is also worth noting that tumor cell dissemination and metastatic colonization does not exclusively rely on changes in cell identity related to the EMT program and its reversal, and other scenarios are also reasonable [15].

## 3. Epithelial-to-Mesenchymal Transition in HNSCC

The expression of well-established markers and key regulators of EMT as well as their potential impact on the pathogenesis and treatment of HNSCC has been reviewed in several recent publications [28]. Further support for the clinical relevance of EMT has been provided by global expression profiling studies, which confirmed the existence of a distinct patient subgroup with a mesenchymal-like gene expression signature in independent HNSCC cohorts [29]. As an example, Keck et al. identified HPV-related and non-HPV-related subgroups with a prominent immune and mesenchymal phenotype [30]. This inflamed/mesenchymal subtype was characterized by a prominent tumor infiltration with cytotoxic T-lymphocytes independent of the HPV status, suggesting a common mechanism to evade immune surveillance by activation of the EMT program. Numerous mechanisms by which cancer cells can escape attack by the immune system have been postulated, and several key regulators of the immune checkpoint control have been identified [31]. The availability of immune checkpoint inhibitors, such as antibodies against PD-L1 (programmed cell death ligand-1), PD-1, and CTLA-4, provides the unique opportunity to revolutionize the treatment of HNSCC [32]. In this context, it is also noteworthy that an increasing body of studies has demonstrated an association between EMT and elevated expression of PD-L1, but also other key regulators of immune checkpoint control in distinct tumor entities, including HNSCC [33]. Consequently, the identification of additional key regulators driving the EMT program could not only pave the way for the establishment of new therapeutic strategies to prevent tumor cell dissemination, but could also improve our understanding of molecular principles modulating the immune checkpoint control.

## 4. The Transcription Factor SOX2

The pluripotency-associated transcription factor SOX2 (sex determining region Y-box 2) is essential during mammalian embryogenesis, adult tissue regeneration, and homeostasis [24]. More recently, SOX2 has been identified as a lineage-survival oncogene in lung and esophageal SCC [34] and recurrent copy number gain of chromosome 3q26; the gene locus encoding SOX2 represents a frequent alteration in HNSCC [35]. As a large body of published studies demonstrates a correlation between high expression levels and poor prognosis, an elevated incidence of recurrence, and/or invasive and metastatic capacity of tumor cells, SOX2 has been considered as a potential therapeutic drug target [36]. However, high SOX2 expression is not uniformly an unfavorable risk factor for survival, and in gastric cancer as well as SCC of the lung and head and neck region, low levels have been reported to correlate with a higher risk for lymphatic metastasis and poor prognosis [37]. These controversial data indicate a high level of context dependency concerning the mode of action during carcinogenesis and the impact of SOX2 on clinical outcome.

**Figure 2:**
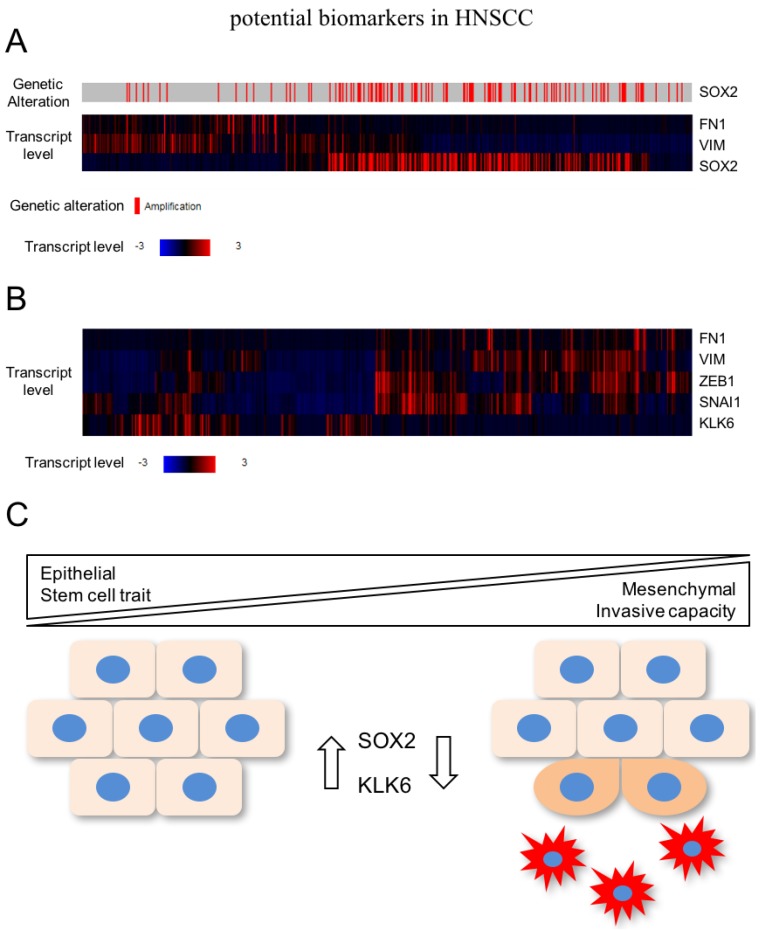
(**A**) Several well-established mesenchymal marker genes (e.g., VIM and FN1) are elevated in primary HNSCC with low SOX2 expression according to TCGA (The Cancer Genome Atlas; https://cancergenome.nih.gov) (**B**) Inverse expression pattern between KLK6 and mesenchymal markers as well as key regulators of EMT in primary HNSCC of the TCGA cohort; (**C**) High expression of SOX2 contributes—among others—to the pathogenesis of HNSCC by promoting tumor cell proliferation. In advanced tumor stages, SOX2 might interfere with tumor cell plasticity and activation of mesenchymal transition by stabilization of the epithelial phenotype including stemness-like traits. KLK6 (kallikrein-related peptidase 6).

In a recent study, global gene expression profiling unraveled a pattern of differentially expressed genes after SOX2 silencing in HNSCC cell lines with 3q amplification, which are related to cell motility, regulation of locomotion, and response to wounding [37]. As described above, these biological processes resemble the activation of EMT, indicating that SOX2 activity stabilizes the epithelial phenotype and prevents mesenchymal conversion of HNSCC cells. In line with this assumption, several well-established mesenchymal marker genes (e.g., VIM and FN1) were up-regulated after SOX2 silencing and are also elevated in primary HNSCC with low SOX2 expression according to public available data from TCGA (The Cancer Genome Atlas; Figure 2A). Moreover, 3q amplification and SOX2 copy number gain is a rare event in the mesenchymal-like subgroup of HNSCC patients [30].

It is also worth noting that for the initiation of somatic reprogramming of fibroblasts into induced pluripotent stem cells, the induction of MET is triggered by SOX2 in a complex with OCT4 [38]. Moreover, the secreted protein CTGF (connective tissue growth factor) has been shown to promote MET in HNSCC cells by inducing c-Jun—a component of the AP-1 transcription factor which activates the transcription of SOX2 and OCT4 [39].

In summary, these data support a model in which high expression of SOX2—as a consequence of copy number variation—but also other so-far less well characterized mechanisms contribute to the pathogenesis of HNSCC by promoting tumor cell proliferation and survival. However, its presence in advanced tumor stages might interfere with tumor cell plasticity and activation of mesenchymal transition by stabilization of the epithelial phenotype, including stemness-like traits (Figure 2C). Although cancer cells with reduced or no SOX2 expression might be more sensitive to systemic treatment with cytotoxic drugs, the mesenchymal-like phenotype potentiates their motility and invasive capacity, which makes an escape from local treatment by surgical resection or radiotherapy more likely. Future studies will be required: (i) to confirm this concept, (ii) to unravel relevant SOX2-related signaling and gene regulatory networks, and (iii) to address the question of whether detection of SOX2-negative tumor cells at the invasive front of primary HNSCC predicts the risk for treatment failure and subsequent loco-regional and/or distant relapse.

## 5. The Kallikrein-Related Peptidase 6

The kallikrein-related peptidase 6 (KLK6) belongs to a family of 15 secreted serine proteases with trypsin or chymotrypsin-like activity, which are encoded by a cluster of genes located on human chromosome 19q13.3–13.4 [25]. Aberrant KLK6 expression is a common feature for many human cancers, and numerous studies evaluated KLK6 as a promising biomarker for early diagnosis or unfavorable prognosis [40]. KLK6 can degrade components of the extracellular matrix, and is implicated in tissue remodeling and the induction of tumor-relevant processes such as proliferation, migration, and invasion [41]. Despite numerous studies pointing to a critical role of KLK6 during neoplastic transformation and malignant progression, more recent data questioned its general tumor-promoting role and stressed the importance of considering its context-dependent function, as exemplified in breast and renal cancer [42].

In the context of HNSCC, a recent study provided experimental evidence that silencing of KLK6 activates the EMT program accompanied by a mesenchymal-like cell morphology as well as accelerated tumor cell migration and invasion [43]. In line with these findings, there is an inverse expression pattern between KLK6 and mesenchymal markers as well as key regulators of EMT in primary HNSCC of the TCGA cohort (Figure 2B). The clinical relevance of these findings was evident by the fact that low KLK6 protein levels in primary HNSCC serve as an unfavorable risk factor for progression-free and overall survival [43]. A critical role of KLK6 in regulating the transition between epithelial and mesenchymal phenotypes was already postulated by Pampalakis et al. [42] They demonstrated that KLK6 acts as a suppressor of tumor progression by promoting MET in breast cancer cell lines, suggesting common mechanisms of KLK6 function in breast cancer and HNSCC cells. However, the underlying mode of action and putative proteolytic downstream targets of KLK6 implicated in EMT and tumor cell plasticity remain to be elucidated.

Concerning the regulation of KLK6 expression, tumor-specific loss in breast cancer is at least in part mediated by epigenetic silencing due to DNA methylation [42]. It will be interesting to address the question of whether a similar mode of regulation also occurs in primary HNSCC with low KLK6 expression. As a consequence, inhibitors of DNA methyltransferases could be used as promising drugs to restore KLK6 expression in order to reverse the EMT program and prevent tumor cell plasticity and dissemination [44].

## 6. EMT-Like Phenotype in Mucosal Melanoma of the Head and Neck

Melanoma is an aggressive tumor arising from melanocytes, endowed with unique features of cellular plasticity. The high degree of phenotypic and functional diversity—which is also a characteristic trait of melanoma cells—is at least in part due to their capacity to reversibly switch between phenotypes with non-invasive and invasive potentials, and is driven by oncogenic signaling and environmental cues [45].

As melanocytes do not express a classic epithelial phenotype, the term EMT cannot be formally attributed to the progression or metastatic spread of melanoma. However, melanocytes exhibit a stable differentiated state via E-cadherin-mediated communication with keratinocytes, while melanoma cells progressively lose E-cadherin in favor of N-cadherin expression [46]. In addition, altered expression of EMT-TFs have been reported in numerous studies, but only recently has the mode of action—how these factors are regulated and orchestrate cellular plasticity in a non-epithelial context (e.g., melanoma)—been addressed in more detail [45]. As an example, Caramel et al. provided a comprehensive overview of the regulatory network of EMT-TFs in cutaneous melanoma with links to oncogenic transformation and tumor cell plasticity [47]. They confirmed prominent SNAIL2 and ZEB2 expression in normal melanocytes, and postulated that both act as tumor-suppressor proteins by activating an MITF-dependent (MITF = Microphthalmia-associated transcription factor) melanocyte differentiation program. However, upon activation of MEK-ERK signaling (e.g., as a consequence of oncogenic NRAS or BRAF mutations), the EMT-TF network undergoes a profound reorganization in favor of TWIST1 and ZEB1. This switch results in E-cadherin loss, enhanced invasion, and constitutes an independent factor of poor prognosis in patients with cutaneous melanoma [48].

Due to the small number of cases of mucosal melanoma patients, there is limited information available on the pathogenesis of this aggressive tumor entity that tends to form local recurrences and regional lymph node metastases in 21%, but a significantly lower number of distant metastases than cutaneous melanoma. Furthermore, there is neither a commercially available cell line for mucosal melanoma nor an established in vivo model for further analysis. Apart from the postulation by Hussein et al. and Dupin et al. that mucosal melanomas arise from melanocytes migrating to non-cutaneous organs after neural crest cells undergo EMT, reliable studies on the expression of classical EMT markers or EMT-TFs in mucosal melanoma are still missing [48].

With regard to the newly identified factors related to an EMT-like phenotype in HNSCC, the expression of KLK6 in cutaneous melanoma was analyzed by Krenzer et al. [41]. Although KLK6 was not detectable in cutaneous melanoma cells, a strong KLK6 protein expression was found in keratinocytes and stromal cells located adjacent to benign nevi, primary melanomas, and cutaneous metastatic lesions. These data suggested a paracrine function of extracellular KLK6 during neoplastic transformation and malignant progression. In our previous work on mucosal melanoma, we characterized MMHN for the expression of KLK6. Paraffin-embedded MMHN of 22 patients were analyzed by immunohistochemical staining, and results were correlated with clinical and pathological data. A positive KLK6 staining was observed in 77.3% (17/22) of MMHN cases, and in line with the situation in HNSCC, a high pattern was significantly correlated with favorable outcome concerning local recurrence-free survival (*p* = 0.013) [7]. The same cohort was used to analyze patterns of PD-L1 expression in MMHN. Interestingly, only 13% (3/23) of mucosal melanoma showed PD-L1 expression, while prominent PD-L1 staining was detected in 100% of tissue sections from a control group of cutaneous melanoma (n = 9) [49]. PD-L1 expression in mucosal melanoma was not correlated with age, sex, nor anatomical localization of the tumor. However, patients with PD-L1-positive mucosal melanoma had a significantly longer recurrence-free survival (*p* = 0.026).

## 7. EMT-Like Phenotype in Salivary Gland Malignancies

Common carcinomas of the salivary glands are classified as adenocarcinoma, mucoepidermoid carcinoma, and adenoid cystic carcinoma (ACC). ACC represents one of the most common malignancies of the salivary glands, and three distinct growth patterns have been identified, which differ in their clinical behavior. The cribriform and the tubular type are usually associated with a better clinical outcome as compared to the solid type of ACC [50]. It is also the most aggressive salivary gland tumor in terms of treatment failure, with a high rate of distant metastases and common perineural invasion [9]. Similar to mucosal melanoma, the availability of reliable preclinical models is limited [51], and most published studies on expression and regulation of EMT-related proteins rely on retrospective studies with FFPE (Formalin-fixed paraffin-embedded) tissue samples. However, reduced expression of E-cadherin in salivary ACCs could be described as compared to paraneoplastic normal salivary tissue [52]. SNAI2 and E-cadherin expression were negatively associated in a cohort of 115 salivary ACC. High SNAI2 and low E-cadherin expression were significantly correlated with perineural invasion [52]. Yi et al. investigated the expression of BMI1—a major component of the polycomb group complex 1 and a candidate stem cell marker—together with EMT-related proteins SNAI1, SNAI2, and E-cadherin in a cohort of 102 ACC patients [53]. They identified a positive correlation between high BMI1 levels and SNAI1 and SNAI2 overexpression. Moreover, high BMI1 levels indicated an unfavorable metastasis-free survival and served as a high-risk marker for salivary ACC. The association between deregulated BMI1 expression and clinical outcome was also observed in an independent study [26]. Elevated expression of the brain-derived neurotrophic factor (BDNF) and its receptor NTRK2 together with reduced E-cadherin expression is a common feature of salivary ACC and significantly correlated with invasion, metastasis, and poor prognosis of ACC patients [27]. The crucial role of the BDNF/NTRK2 axis was further supported by a more recent study demonstrating that NTRK2 levels are positively correlated with expression of the EMT-related protein S100A4 but negatively associated with E-cadherin levels [54]. Both NTRK2 and S100A4 were positively associated with perineural invasion, indicating that specific targeting of the BDNF/NTRK2 axis might represent a promising new therapeutic strategy for ACC patients.

## 8. Conclusions and Outlook 

Tumor cell dissemination as enabled by EMT and followed by MET has been considered as a hallmark of metastasis [55]. However, alternative modes of dissemination, such as collective or cluster-based migration and invasion can exist and may not even exhibit an overt up-regulation of mesenchymal markers. Furthermore, disseminated cancer cells may undergo metastatic colonization via an MET-independent pathway. Together, the wealth of data acquired thus far support a more nuanced view of the role of EMT and MET in cancer metastasis. While in some cases these programs are critically important, in other scenarios EMT and MET may not play an important role, but more of permissive and potentially catalytic roles by regulating phenotypes that accelerate the processes necessary to escape and colonize [14]. Moreover, recent reports investigating circulating cancer cells in the bloodstream or employing genetic lineage-tracing have questioned a critical role of an EMT in metastasis formation. Hence, we need to better understand the molecular networks underlying the cell plasticity conferred by an EMT or a MET and its functional contribution to malignant tumor progression. Although the exact mechanisms of EMT remain complex, certain aspects and connections have become more evident. The loss of KLK6 in HNSCC seems to create a mesenchymal-like morphology and accelerated motility of tumor cells with an EMT-phenotype, identified by loss of E-cadherin and prominent induction of vimentin expression in HNSCC cells. The clinical relevance of these findings is supported by the fact that low KLK6 protein level in primary HNSCCs serves as an unfavorable risk factor for progression-free and overall survival [43]. Additionally, it has been shown that not only the loss of KLK6 but also the loss of SOX2 expression induces cell motility via vimentin up-regulation and is an unfavorable risk factor for survival of head and neck squamous cell carcinoma [37]. Low SOX2 expression seems to be an unfavorable risk factor for poor clinical outcome, and serves as a prognostic marker to identify HNSCC patients with high risk for treatment failure due to an invasive phenotype. On the other hand, KLK6 in MMHN seems to be highly overexpressed, but also correlated with a better clinical outcome [7]. Although the accuracy of this statement must be questioned given the small number of MMHN cohorts, the protective role of KLK6 has been described before in other tumor entities such as breast cancer, and therefore seems to be highly dependent on the microenvironment and the tumor entity. In contrast to previous results in HNSCC, SOX2 seems to promote invasion and tumor progression by creating a more mesenchymal phenotype in ACCs [56]. These controversial findings of well-described mesenchymal and epithelial markers as well as new mediators of EMT in three tumor entities with different progressive behavior underlines that the exact molecular mechanisms underlying the cross-linking between various EMT pathways in different HNCs remains to be fully elucidated. The main difficulty seems to be the quantification of “partial EMT” in each disease state and capturing these dynamic states. Partial EMT phenotypes reflect the enhanced plasticity of tumors and are important for understanding the progression of EMT and MET processes. However, this “moving target” presents a major challenge in rational drug design. Nevertheless, with fresh knowledge and the benefit of hindsight, certain principles have emerged. The exploration of EMT and MET mechanisms is destined to pave the way for future clinical implications. EMT markers could not only serve as biomarkers for chemotherapy response and survival prognosis, but also represent a targetable process, aiming to prevent metastasis and overcoming drug resistance. In different solid cancers, EMT has been linked to a generation of cancer cells with stem cell attributes of tumor initiation and resistance to chemotherapy [57]. In clinical tumor samples, EMT has been implicated with the acquisition of drug resistance. Moreover, the inhibition of EMT signal cascades by specifically tailored drugs has been tested in vivo. As a result, miR-506 sensitized EOC (epithelial ovarian cancer cells) to chemotherapy and inhibited EMT-mediated metastasis [58]. The group of Chiu et al. described FOXM1 as a critical regulator of an EMT, and were able to show that the combination of a FOXM1 inhibitor and cisplatin led to increased expression of EMT-related markers in chemoresistant cells [59]. In contrast to classical chemotherapy, these experimental approaches towards targeting EMT cascades in cancer aim to diminish or even abolish metastasis formation without doing harm to healthy cells.

In summary, we are facing an urgent need to characterize the distinct cellular states associated with EMT plasticity to identify targetable EMT components and create therapeutic strategies to effectively eliminate cells undergoing EMT, since this is ultimately leading to treatment failure. However, by further analyzing the already existing results and investigating the pathogenesis of EMT in different head and neck cancers, the knowledge on cellular and molecular principles of malignant progression can be improved, ultimately aiming to stratify cancer patients at high risk for poor therapy response and to provide applicable therapies targeting EMT processes. By exploring the complex underlying mechanisms which lead from tumor cell dissociation in the primary tumor through EMT towards the formation of metastasis (including MET), our findings might offer promising perspectives for the future.

## Figures and Tables

**Table 1:** Expression of different molecules and their participation in biological behavior and epithelial-to-mesenchymal transition (EMT). HNSCC: Head and neck squamous cell carcinoma.

**Established EMT-Markers**	**Biological Behavior **	**Role in EMT/Tumorigenesis**	**Reference**
Vimentin	Type III intermediate filament that is found in mesenchymal cells of various types	Marker of cells undergoing an epithelial-to-mesenchymal transition (EMT) during both normal development and metastatic progression	[16]
E-cadherin	Protein encoded by the CDH1 gene, also been designated as CD324, tumor suppressor gene	Loss of E-cadherin function/expression is implicated in cancer progression/metastasis, downregulation decreases the strength of cellular adhesion, resulting in an increase in cellular motility	[17]
N-cadherin	In embryogenesis, N-cadherin is the key molecule during gastrulation and neural crest development	Promotes tumor cell survival, migration, and invasion, and high levels are often associated with poor prognosis	[18]
Fibronectin	Many different cells are capable of incorporating plasma fibronectin into their extracellular matrix of any tissue	Cancer-associated fibroblasts (CAFs) are essential sources of increased extracellular matrix deposition and altered remodeling to pave the way for cancer cell invasion.	[19]
SNAI1/2	Snail superfamily of zinc-finger transcription factors, involved in cell differentiation and survival. Snail1: essential for gastrulation. Snail2: embryonic development	Snail1: Common sign of poor prognosis in metastatic cancer, and tumors with elevated Snail1 expression show high rates of treatment failure	[20]
		Snail2: Tumor metastasis promotes EMT through activation of SNAIL2 in HNSCC	
TWIST1	Helix-loop-helix transcription factor, plays an essential and pivotal role in multiple stages of embryonic development	Promotes the formation of cancer stem cells and EMT, targeting TWIST1-related molecules significantly inhibits tumor growth and thus improves the survival of cancer patients	[21]
ZEB1/2	Zinc finger E-box binding homeobox 1/2, acts as transcriptional repressor	Dual role: (1) repressor for epithelial genes. (2) a transcriptional activator when associated with YAP (Hippo Pathway); also known to induce EMT in various cancers, but has also been linked to promote treatment failure in an EMT-independent manner	[22]
**Markers Associated with EMT**	**Biological Behavior**	**Role in EMT/Tumorigenesis**	**Reference**
PD-L1 and PD-1	PD-L1: cluster of differentiation 274(CD274) or B7 homolog 1 (B7-H1), 40kDa type 1 transmembrane protein, plays a role in suppressing the immune system during pregnancy, tissue allografts, autoimmune disease, and others, ligand of programmed cell death protein-1 (PD-1). PD-1: Cell surface receptor that plays a role in promoting self-tolerance by suppressing T cell inflammatory activity	Many tumor cells express PD-L; inhibition of the interaction between PD-1 and PD-L1 can enhance T-cell responses in vitro and mediate preclinical antitumor activity. This is known as immune checkpoint blockade	[23]
SOX2	Pluripotency-associated transcription factor SOX2 (sex determining region Y-box 2), essential during mammalian embryogenesis, adult tissue regeneration, and homeostasis	Identified as a lineage-survival oncogene in lung and esophageal SCC and recurrent copy number gain of chromosome 3q26, the gene locus encoding SOX2 represents a frequent alteration in HNSCC	[24]
KLK6	Kallikrein-related peptidase 6 (KLK6), Family of 15 secreted serine proteases with trypsin or chymotrypsin-like activity, encoded by a cluster of genes located on chromosome 19q13.3–13.4	Common feature for many human cancers, promising biomarker for early diagnosis or unfavorable prognosis. KLK6 can degrade components of the extracellular matrix and is implicated in tissue remodeling and induction of tumor-relevant processes such as proliferation, migration, and invasion	[25]
BMI1	BMI1 (B lymphoma Mo-MLV insertion region 1 homolog) has been reported as an oncogene by regulating p16 and p19	BMI1 deregulation is associated with enhanced migration, invasion, and poor prognosis in salivary adenoid cystic carcinoma	[26]
BDNF	Brain-derived neurotrophic factor (BDNF) acts on certain neurons of the central and the peripheral nervous system, helping to support the survival of existing neurons and encourage the growth and differentiation of new neurons and synapses	Elevated expression of the brain-derived neurotrophic factor (BDNF) and its receptor NTRK2 together with reduced E-cadherin expression is a common feature of salivary adenoid cystic carcinoma (ACC) and significantly correlated with invasion, metastasis, and poor prognosis of ACC	[27]
NTRK2	Receptor tyrosine kinase involved in the development and maturation of the central and the peripheral nervous systems through regulation of neuron survival, proliferation, migration, differentiation, and synapse formation and plasticity	NTRK2 levels are positively correlated with expression of the EMT-related protein S100A4 but negatively associated with E-cadherin levels	[27]
